# WHAT ROLES FOR TRACK-STRUCTURE AND MICRODOSIMETRY IN THE ERA OF -omics AND SYSTEMS BIOLOGY?

**DOI:** 10.1093/rpd/ncy221

**Published:** 2018-12-11

**Authors:** G Baiocco, G Babini, S Barbieri, J Morini, W Friedland, C Villagrasa, H Rabus, A Ottolenghi

**Affiliations:** 1 Physics Department, University of Pavia, Pavia, Italy; 2 Institute of Radiation Protection, Helmholtz Zentrum München – German Research Center for Environmental Health, Neuherberg, Germany; 3 Institut de Radioprotection et Sûreté nucléaire (IRSN), Fontenay aux Roses Cedex, France; 4 Physikalisch-Technische Bundesanstalt (PTB), Braunschweig, Germany

## Abstract

Ionizing radiation is a peculiar perturbation when it comes to damage to biological systems: it proceeds through discrete energy depositions, over a short temporal scale and a spatial scale critical for subcellular targets as DNA, whose damage complexity determines the outcome of the exposure. This lies at the basis of the success of track structure (and nanodosimetry) and microdosimetry in radiation biology. However, such reductionist approaches cannot account for the complex network of interactions regulating the overall response of the system to radiation, particularly when effects are manifest at the supracellular level and involve long times. Systems radiation biology is increasingly gaining ground, but the gap between reductionist and holistic approaches is becoming larger. This paper presents considerations on what roles track structure and microdosimetry can have in the attempt to fill this gap, and on how they can be further exploited to interpret radiobiological data and inform systemic approaches.

## INTRODUCTION

When investigating the mechanisms and outcome of the radiation insult to a cell, as the basic structural and functional unit of a living organism, one is confronted with a peculiar kind of perturbation: ionizing radiation action proceeds through stochastic, discrete energy deposition events (excitations or ionizations), occurring over a very short temporal scale (up to 10^-15^ s) and a spatial scale such that critical cell targets as the nuclear DNA (double helix diameter ~2 nm) and chromosomes (linear dimension of a domain ~1 μm) can be severely damaged^(1, 2)^. As widely recognized, the biological outcome of the exposure strongly depends on the clustering of energy deposition events at such scales, in turn related to the radiation quality under investigation (i.e. densely vs. sparsely ionizing radiation)^(3)^. This largely accounts for the success of mechanistic approaches in radiobiological research, as track structure (and nanodosimetry) and microdosimetry studies, though often limited to the characterization of energy deposition, initial damage and its complexity: the further correlation of these quantities to late radiobiological endpoints usually requires a phenomenological modeling approach, and the intrinsic variability in energy deposition at the target level is not always taken into account (e.g. when extracting only the average of microdosimetric distributions and not their shapes).

The knowledge of the initial characteristics of the perturbation is necessary but not sufficient to fully understand the complex reaction of a biological system, particularly when dealing with responses at the supracellular organizational level (from tissues and organs, to systems and apparatuses) and involving long time periods, as for the induction of radiation-induced cancer^(4)^ or cardiovascular diseases^(5)^. Besides DNA damage (direct or mediated by free radicals), the whole cell machinery is affected by radiation, and other cellular components may be involved in the cascade of events initiated by radiation, without necessarily being initial targets. It is becoming increasingly apparent that at low dose and dose rate DNA is not the primary target for radiation damage, and the stochastic effects of radiation that may become apparent over a time frame of hours to tens of years are not primarily associated with classical mutagenesis^(6)^. Radiation-induced perturbation of cell signaling plays a major role in mechanisms at the basis of the so-called non-targeted effects and in determining the characteristics of the cell environment, as in the case of an inflammatory response. How the system copes with the initial damage is also subject to great biological variability, depending on factors such as the cell cycle phase of hit cells, epigenetic factors, individual radiosensitivity due, e.g. to the presence of mutations affecting DNA repair pathways and other aspects of genetic background, as the presence of different polymorphisms. This view of the biological target as a system or network of interacting components calls for holistic instead of reductionist approaches to fully understand the mechanisms behind the biological response to radiation. Systems biology^(7)^ studies, addressing all different levels in a cell, e.g. from the DNA (genome), RNA (transcriptome) to protein pool (proteome) and metabolite pool (metabolome), and how these are possibly affected by radiation, are increasingly gaining ground. Bioinformatics analysis of vast -omics datasets, together with the development of new methods to perform an integrated analysis of different radiobiological endpoints, are promising to gain further insight into these mechanisms.

Taken altogether, these considerations beg the following questions: what roles for track structure and microdosimetry, and, more in general, for the study of radiation-induced initial events, in radiobiology today? And furthermore: what strategies can be adopted to fill the gap between a reductionist approach, based on the initial characterization of radiation-induced energy deposition in a specific target, at the highest level of detail, and a holistic approach, delivering a picture of the whole cell and all its interacting components ‘at a glance’, also a long time after the exposure?

Bearing these questions in mind, this paper starts with a first review of recent results obtained with track structure and microdosimetry approaches, followed by a discussion of their implications, and it finally gives indications on possible strategies to make the bridge towards systemic approaches in radiation biology.

## TRACK-STRUCTURE AND MICRODOSIMETRY APPROACHES

This section includes selected examples of track structure and microdosimetry calculations that, to some extent, present aspects of novelty, and might point a way forward for the application of such approaches to gain new insight for the interpretation of radiobiological data.

### TRACING BACK RADIOBIOLOGICAL EFFECTS TO THE PHYSICS

Going back to the physics often offers the best chance of interpreting radiation quality effects, namely the fact that the same amount of energy delivered to a biological target by different kinds of radiation leads to very different biological consequences. This is particularly evident when dealing with exposures to a mixed field of radiation (e.g. for the secondary charged particle field induced by neutrons). The variation of neutron relative biological effectiveness (RBE) as a function of neutron energy has been recently explained based on a coupling of neutron transport and track structure calculations for neutron-induced secondary charged particles^(8)^. The proposed *ab initio* neutron RBE model is based on the induction of DNA double strand break (DSB) clusters, i.e. clusters of two or more DSBs within a genomic distance of 25 bp. Predicted RBE values show a good qualitative agreement with the energy dependent radiation weighting factors (*w*_R_) for neutrons, established by ICRP 103 based on a variety of radiobiological endpoints. What is interesting is that, though certainly not exhaustive of all endpoints analysed to set the *w*_R_ function, complex DNA damage (as an indicator of radiation clustering properties) remains a powerful tool to investigate correlations with the biological outcome and how such outcome varies as a function of the field characteristics. An alternative neutron RBE model was also presented in the same work^(8)^, based on the dose mean lineal energy in a sensitive spherical site of 1 μm diameter. Also with this model, a good qualitative agreement with the *w*_R_ function given for neutrons in ICRP 103 is obtained, though input from data is needed to reproduce the saturation of the biological effect with increasing linear energy transfer (LET).

It is also worthwhile to notice that contradictory results are found in the literature on whether neutron-induced bystander effects exist^(9, 10)^. As energy depositions in the target are strongly dependent on neutron field characteristics and target geometry, the interplay between neutron-induced targeted and non-targeted effects might vary for different neutron fields. This makes the formulation of a general conclusion improper, and calls both for a full description of the radiation field at the point of interest and for a detailed study of the link between energy deposition and signalling cascades, to unambiguously interpret results on neutron-induced bystander effects.

As track structure approaches offer the highest level of detail for the characterization of the spatial distribution of energy depositions, their predictions can play an important role when dealing with the biological effect of internal emitters. Energy deposition in any finite track-segment or target size can be derived based on Monte Carlo track modeling. Any desired cellular geometry can be implemented, also dependent on culture conditions for *in vitro* measurements, as well as any distribution of radionuclides in cell compartments (cytoplasm, nucleus, cell surface, etc.). Recent calculations of track-structure based *S*-values (dose to the target nucleus per source decay) for electrons as a function of their energy led to differences with respect to the MIRD standard for use in dose calculations for research and applications in nuclear medicine^(11)^. Though beyond the focus of this paper, it has to be mentioned here that differences in predictions of nano- and microdosimetric quantities are also observed when using different calculation tools, and this deserves attention through dedicated intercomparison efforts^(12)^ to provide reliable results for further analysis.

Finally, track structure and microdosimetry approaches are certainly suited to addressing the enhancement of biological effects that can be traced back to a physical origin (although a chemistry-driven enhancement might be prevailing in some cases), as it is the case for X-ray exposure of target cells doped with gold nanoparticle^(13)^, or proton/neutron irradiation of cells with boron compounds^(14)^.

### SIMULATING THE ‘OBSERVER’

Track-structure studies are of great usefulness when trying to go ‘behind’ experimental data, simulating the observer to overcome the limitations of experimental techniques, thus gaining insight on the induction of biological damage whose characteristics are unmeasurable (at least with standard techniques). Remarkable examples in this sense are the prediction of the yield of small chromatin fragments in the case of chromosome aberrations^(15)^ or of very short DNA fragments when investigating how the DNA fragmentation pattern varies with radiation quality^(16)^. Track-structure approaches coupled to a realistic description of the DNA target can be used for the investigation of damage characteristics behind radiation-induced foci^(17)^. Notably, a saturation of the signal of γ-H2AX foci as a function of increasing dose is observed experimentally, also for low LET reference radiation, occurring at a dose dependent on the readout technique^(18)^. This might render the RBE concept intrinsically not applicable to this endpoint. What is measured in standard immunocytochemistry protocols with 2D microscopy is the yield of foci that can be counted in a projected image of a cell nucleus slice, selected as a result of optical focusing, with a resolution limit dictated by the size of the smallest detectable focus. Experimental results can then be compared to what is obtained starting from a track-structure based prediction of the 3D spatial distribution of damages in the whole cell nucleus, when the readout is also simulated^(18)^, thus obtaining a quantification of the actual damage behind observations (also carefully considering the time dynamics of foci induction and disappearance).

### CHANGING THE INITIAL TARGET

There is potential for extending track structure approaches to initial targets other than nuclear DNA. Damage to mitochondrial DNA (mtDNA) has recently been simulated for different radiation qualities (^60^Co γ’s and α particles)^(19)^. An *in silico* replica of mtDNA has been developed. Energy deposition to cell mithocondria was found to be highly inhomogeneous, especially at low doses. Whilst little damage to mtDNA occurs, even at large doses, evidence is accumulating of metabolic damage to mitochondria associated with oxidative stress^(20)^, particularly at doses associated with non-targeted effects. Major perturbations to mitochondrial membrane potential^(21)^, gene expression and enzyme activity^(22)^ occur, but the molecular target, whether protein, RNA or lipid for example remains unknown, and mitochondria appear to be key players in radiation damage at medium to low dose.

Besides mitochondria, other cell components should be tested as possible initial targets of radiation damage, as e.g. the nuclear membrane, the endoplasmic reticulum, ribosomes, etc. Besides what happens in organelles, also energy depositions in a variety of macromolecules (proteins, lipids, carbohydrates, etc.) could be characterized as a function of radiation quality, though it can be expected that most are involved in the response to radiation as players in cellular function, and not necessarily as initial targets.

The implementation of cell models with specific morphology in a track structure simulation environment can also provide new information on tissue-specific biological effects: efforts of this kind are currently being undertaken e.g. for the investigation of space radiation effects to the central nervous systems. Heavy particles in a space radiation environment give rise to tracks with a highly heterogeneous energy deposition pattern. Using *ad hoc* developed *in silico* neuron models^(23)^, the spatial dose distribution to specific neuronal components can be calculated, leading to different structural damages and biological outcome. This could be extended to other cases of cells with morphological and functional specificities, or in principle, to cell aggregates with specific cellularity and/or protein distribution.

## TOWARDS A SYSTEMIC APPROACH

Systems approaches can be distinguished in two categories^(24)^: bottom-up approaches, trying to reconstruct the interacting network from its components, and top-down approaches, focusing on the system as a whole, with the aim of highlighting relevant pathways and the underlying non-linear interactions, to be further analyzed in detail.

A practical application of a bottom-up approach is the integration of different radiobiological endpoints in a common analysis, when, for example, such endpoints are measured on the same biological system (e.g. a cell line in a specific experimental setup), and specific comparisons are made possible depending on experimental conditions (e.g. isodose or isotime comparisons), eventually allowing to identify and validate biomarkers on different *in vitro* and *in vivo* models. Track-structure or microdosimetry approaches could provide results to be included in the integration: either quantities to be directly benchmarked with data (if the simulation of the experimental readout for the endpoint is possible) or quantities to be used for correlation analysis.

Following a top-down approach, integrated-omics datasets might highlight radiation-perturbed molecular pathways and/or associated cellular substructures. The key players of these pathways need to be investigated through temporal and spatial dynamics. Track-structure or microdosimetry approaches can be used to characterize energy depositions in macromolecules found to be of relevance in the identified pathways. If the role of such macromolecules as initial target of radiation is easily ruled out, then the link between energy deposition and functioning/signaling must be further investigated.

As a general strategy, we can possibly use systemic approaches to have ‘a hint’ on where to look at when the system is perturbed by radiation, and then resort to ‘sniper-rifle’ approaches to gain knowledge on the involved sub-systems and their functioning. Among these latter, track structure and microdosimetry approaches are useful for the interpretation of radiobiological data, and can inform a systemic description of the biological response to radiation. This remains particularly true when comparing the outcome of the exposure to different radiation qualities, whose effectiveness is ultimately dependent on the spatial pattern of energy depositions caused by radiation, that can be characterized at best with such approaches.
